# An Indexing Method of Continuous Spatiotemporal Queries for Stream Data Processing Rules of Detected Target Objects

**DOI:** 10.3390/s21238013

**Published:** 2021-11-30

**Authors:** Muhammad Habibur Rahman, Bonghee Hong, Hari Setiawan, Sanghyun Lee, Dongjun Lim, Woochan Kim

**Affiliations:** 1 Department of Computer Science and Engineering, Pusan National University, Busan 46241, Korea; Mhabiburr17@pusan.ac.kr (M.H.R.); 960416-5120063@pusan.ac.kr (H.S.); geodb@pusan.ac.kr (S.L.); dannylim0709@pusan.ac.kr (D.L.); 2 Agency for Defense Development, Changwon 34186, Korea; woochankim@add.re.kr

**Keywords:** continuous query, index, rule processing, spatiotemporal query, stream data

## Abstract

Real-time performance is important in rule-based continuous spatiotemporal query processing for risk analysis and decision making of target objects collected by sensors of combat vessels. The existing Rete algorithm, which creates a compiled node link structure for executing rules, is known to be the best. However, when a large number of rules are to be processed and the stream data to be performed are large, the Rete technique has an overhead of searching for rules to be bound. This paper proposes a hashing indexing technique for Rete nodes to the overhead of searching for spatiotemporal condition rules that must be bound when rules are expressed in a node link structure. A performance comparison evaluation experiment was conducted with Drool, which implemented the Rete method, and the method that implemented the hash index method presented in this paper. For performance measurement, processing time was measured for the change in the number of rules, the change in the number of objects, and the distribution of objects. The hash index method presented in this paper improved performance by at least 18% compared to Drool.

## 1. Introduction

**Figure 1:**
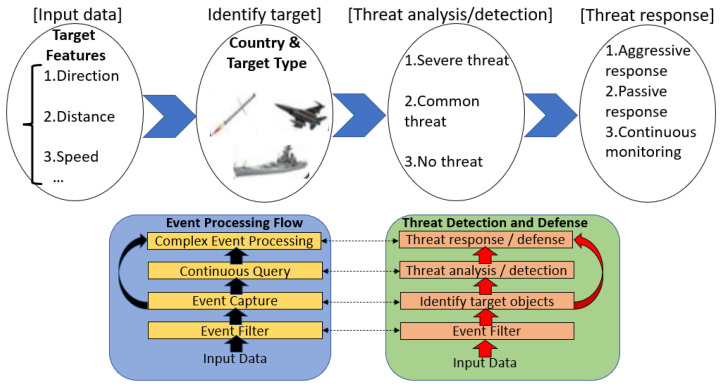
Rule-based event processing framework.

A ship combat system uses a rule-based event processing application for threat analysis and weapon response [1,2] for target objects collected in real time. As shown in Figure 1, it is assumed that the target object data collected from the radar sensor, sonar sensor, and electromagnetic sensor [3] installed on the ship are ID, speed, elevation, IFF, direction, location, and time; here, ID is the identifier of a target object, and IFF is the foe or friendly identification information. The input data in Figure 1 are stream data collected from the data distribution service topic [4]. Threat analysis and decision making are performed by applying rules for event processing, event capturing, continuous query (CQ) [5,6,7,8] processing, and complex event processing [9,10,11,12,13,14,15,16]. For complex event processing, the operator scheduling method [17] that uses a similarity-based preference was proposed to address the difficulty in determining complex events due to the uncertainty of dynamic input events; however, it is still not suitable to address the processing performance of rule binding. The continuous query repeatedly executes a given query condition for each stream of data that is continuously input in real time.

The performance of the rule-based event processing framework is determined by the search time to look for some appropriate rules to the stream input data in real time and the processing time of individual rules. The set of rules of event filter, event capture, CQ, and complex event processing [18,19,20,21] are interrelated. Thus, rules should be executed as a binding process in which the output of one rule becomes the input of another rule. A complex event implies that when at least two events are input to a rule, join events that satisfy the rule condition must be generated. The rule execution by interpretation presents a slow processing speed when stream data are input. To alleviate this problem, a Rete algorithm, which means network in Italian, was proposed to maintain the internal data structure by complying with the rules [22].

When there are *N* target moving objects that are continuously collected in real time, the rule processing for *M* continuous query rules should be repeated *N* × *M* times. Usually, the detection of the target objects by the radar is repeated every 3 to 5 s. Let *p* be the number of times the target stream objects are collected within a given period. If the input of stream data of target objects continuously collected is repeated *p* times, the total number of rule processing becomes *N* × *M* × *p*, and then, the time complexity becomes approximately *O(n^3^)*. Therefore, even if the compiled internal data structure of the rule using the Rete algorithm is used, the performance significantly degrades. In particular, in the case of a rule including a spatiotemporal CQ condition, the performance of rule binding becomes worse.

Existing studies incorporated Java topology suite-based spatial functions [23,24] to ensure the inclusion of spatial data processing functions in rules by extending the Rete algorithm. The extension of the Rete algorithm to support spatial functions in the processing of conditional operation of rules was implemented in the Drools engine [25]. However, the stream data processing method of the improved Rete algorithm was required to examine all the rules for input data consecutively. Hence, the rule processing performance was not efficient when the number of rules was significant. To reduce unnecessary join operations of beta nodes in the condition processing of rules, the concept of the time-stamped event to reduce join targets using time constraints was proposed [26]. An alternative method, which is a performance improvement technique, was also proposed to reduce the search overhead of rules by applying a hash map to the Rete root node and classifying it as a FactTypeNode [27,28].

However, existing studies on the performance improvement of rule-processing based on the extension of Rete algorithm did not consider the index for rules, including spatial conditions. The existing studies failed to address the problem of performance deterioration caused by an increase in the number of rules. Therefore, this study proposes a performance improvement algorithm [29] that can instantaneously determine the rule by stabbing the stream event input in real-time using a pre-made hash index for the spatiotemporal query conditions. The CQ conditions of the rule were implemented using the spatial index of the R* Tree [30].

This study addresses the problem of performance degradation when the number of alpha nodes bound to beta nodes increases when alpha nodes are continuous spatial queries in the Rete node structure. We propose a hash CQ index for two or more alpha nodes bound to beta nodes in the Rete node network of bound rules. This study aimed to reduce the number of rules that need to be searched by CQ indexing, by creating a spatiotemporal index for the nodes generated by the Rete algorithm. This study makes the following contributions. An algorithm is proposed that determines the binding rule to a better node through a stabbing process for events that are dynamically continuously input by creating a CQ index when the alpha node bound to the bettor node is spatiotemporal CQ. To evaluate the hash CQ index and stabbing algorithm presented in this paper, we conducted a performance evaluation experiment with Drool, which has commercialized the existing Rete algorithm, according to the change in the number of rules and the number of target objects.

The remaining paper is structured as follows: Section 2 examines the continuous query rule processing and addresses the problem. Section 3 presents the algorithm details to solve the addressed problem. Section 4 exhibits the performance evaluation results, and Section 5 draws the concluding remarks of the article.

## 2. Spatiotemporal Continuous Query Rule Processing

This section examines the Rete structure compiled by continuous and complex event processing rules.

### 2.1. Stream Processing Rules of Target Objects

**Figure 2:**
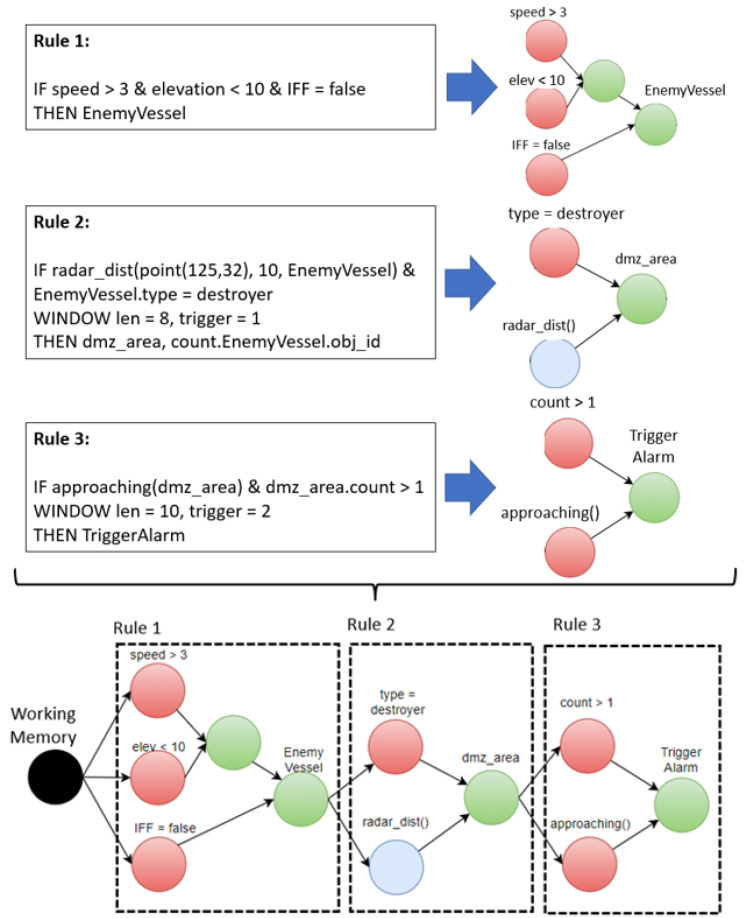
Rete node network for bounded rules.

The Rete algorithm [22] incorporates Rule1, Rule2, and Rule3 shown in Figure 2 into a network of nodes composed of alpha nodes (red) and beta nodes (green). A beta node includes a join operation between the alpha nodes or between beta nodes. As shown in Figure 2, the beta node was used as an input to the alpha node of the bound rules. The alpha node (blue), including the CQ operation of Rule2, received and processed the event from the beta node of Rule1. The red alpha node of Rule1 represents a condition for a simple literal condition, and the blue alpha node of Rule2 represents a spatiotemporal condition.

**Figure 3:**
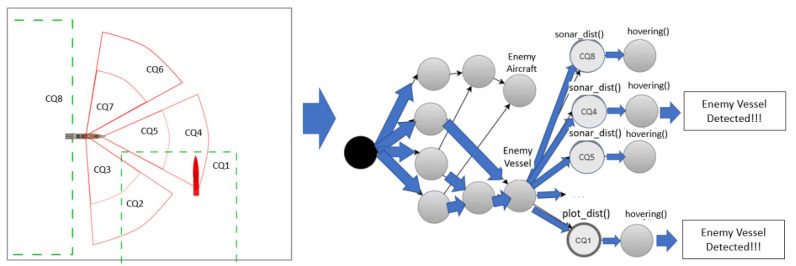
Example of Rete network created for eight continuous query rules.

When the output value of a beta node was input to more than one alpha node, the corresponding alpha nodes were executed sequentially. In particular, when the alpha node was a continuous spatial query, the performance decreased because the corresponding spatial operation was executed sequentially for each rule. As shown in Figure 3, when there were eight continuous spatiotemporal queries defined for one vessel, eight alpha nodes connected to a given beta node were created to perform CQ operations. The CQs entered by the beta node ‘Enemy Vessel’ are CQ1 and CQ4. Rather than searching for CQ1 to CQ8 bound to beta nodes one by one, we propose a method of stabbing search by creating a spatiotemporal index for continuous queries. In Figure 3, EnemyVessel is a beta node, and CQ1–CQ8 are alpha nodes.

### 2.2. Spatiotemporal Continuous Query Rules

In the Rete node network of bound rules, the hash index for the alpha node, including the CQ condition, was used to select the alpha node to apply to the beta node. As shown in Figure 2, EnemyVessel was created as a beta node of Rule 1, and EnemyVessel was used as the CQ condition of Rule 2. Therefore, the CQ condition of Rule 2 was an alpha node. This study proposed a new method for determining the alpha node of Rule 2 as a hash index for a stream event EnemyVessel to satisfy the spatiotemporal condition of Rule 2 [31]. When the alpha node of the CQ condition was converted into a spatiotemporal index, the stream event of the beta node became a stabbing process for the spatiotemporal CQ index, thereby reducing the search overhead of the rules [29].

**Figure 4:**
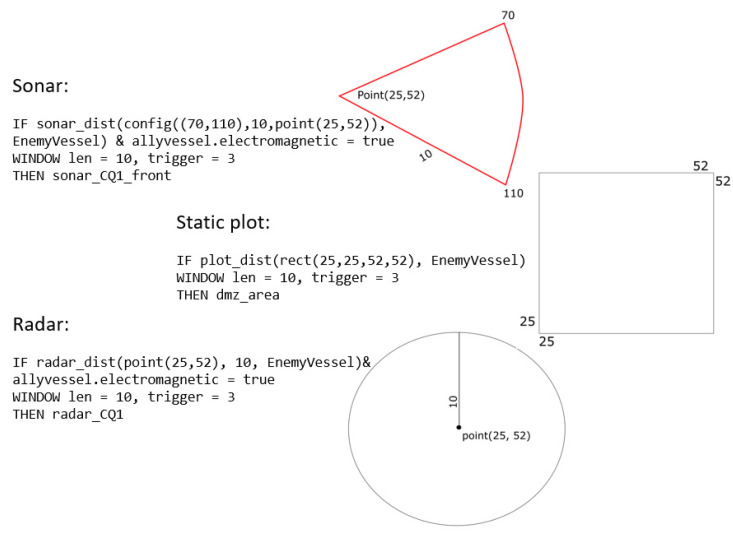
Rule examples with spatiotemporal CQ conditions.

The spatiotemporal condition was expressed as a polygonal area with a fixed position, or a sector or circle set by the radar or sonar sensor range of the ship, as shown in Figure 4. When the vessel moved, the sectoral or circular space-time condition was expressed as a spatiotemporal condition because the position of the space condition changed with time. As shown in Figure 4, when a rule including a spatial condition for the beta node EnemyVessel was added, it was processed as an update of the continuous query index. When the spatial CQ rule is added, as shown in Figure 4, an alpha node bound is added to the beta node shown in Figure 3.

## 3. Rete Node Indexing and Stabbing Algorithms

This section describes the algorithm for inserting the hash CQ index when the spatial CQ rule is added as well as the stabbing process algorithm for alpha nodes.

### 3.1. Hashing Index of Rete Node

**Figure 5:**
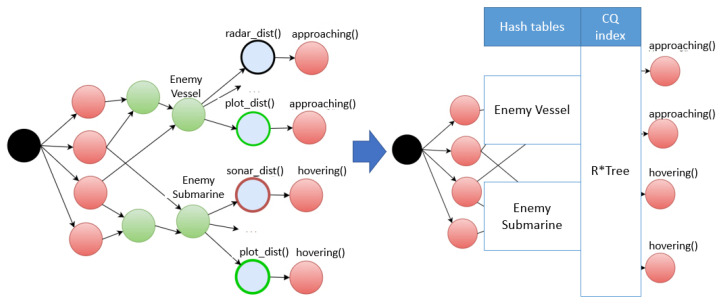
Examples: introduction of hash indexes for beta nodes.

The first Rete algorithm was designed to improve the performance of rule processing for logical reasoning in static databases [22]. The improved method of the existing Rete algorithm [32] proved to be effective for stream event processing; however, the overhead of processing all rules sequentially to process input stream data continuously remains unsolved. In the Rete structure shown in Figure 5, EnemyVessel and EnemySubmarine are beta nodes, and there is a time-wasting factor to sequentially execute alpha nodes to express the CQs. Figure 5 shows an example of introducing a hash index to beta nodes to manage the output value of the beta node in the hash table and reduce the rule search overhead with the stabbing algorithm by creating an index in the CQ space. The code written in italics in Algorithm 1 is the process of creating an index for a spatial node. In addition to the existing Rete algorithm, a spatial node index construct processing process was developed.
**Algorithm 1** Rule Insertion (r)1 **Input:** vector<string> Input //string of rule input; 2 **Output:** RETE net and updated Node Indexing;   /* Based on node identification, build each node*/ 3 expVec = new vector<pair<string,string>> //node type and rule input; 4 While (Input != end) { 5  extracted=DecomposeRule(Input[i]); 6  expVec.push(extracted); 7 } 8 nodeVec = new vector<Node*> //created node; 9 scalarVec = new vector<Node*> //created CQ node; 10 spatialVec = new vector<Node*> //created Spatial node; 11 While (expVec != NULL && nodeVec.size()—1){ 12  If (expVec.first == “Alpha”) { 13   tempAlphaNode = new AlphaNode(expVec.second); 14   nodeVec.push(tempAlphaNode); 15   scalarVec.push(tempAlphaNode); 16   expVec.pop(); 17  } 18  Else {   /* check whether the previous node is an existing node */ 19   If (expVec.first == “spatial”) { 20    tempBetaNode = new BetaNode(expVec.second); 21    nodeVec.push(tempBetaNode); 22    spatialVec.push(tempBetaNode); 23    expVec.pop() 24   } 25   Else { 26    If (isExist(expVec.second)) { 27     nodeVec.push(findNode(expVec.second)); 28    } 29    Else { 30      tempBetaNode = new BetaNode(expVec.second); 31      nodeVec.push(tempBetaNode); 32     } 33     expVec.pop(); 34    } 35   } 36   tempBetaNode = newBetaNode(nodeVec.top(), nodeVec.top()-1); 37   connectNode(nodeVec.top, nodeVec.top()-1, tempBetaNode); 38   nodeVec.pop(); 39   nodeVec.pop(); 40   nodeVec.push(tempBetaNode); 41  } /*Spatial Node Index Construction */ 42  *While (spatialVec.size() > 0) {* 43   *pointVec = Utilities::decomposeNode(spatialVec.top);*   /*Get the entity name from the CQ node */ 44  *observedEntityName = getEntityName(spatialVec.top);*   /*from the existing node indexing tree, insert the CQ area */ 45  *(spatialIndex[observedEntityName]).insert(pointVec);   /*remove the node from the vector */ 46   *spatialVec.pop();* 47  *}*


In the study of a typical implementation and application of the Rete algorithm [19], alpha nodes and beta nodes are constructed by compiling the rules input as text and subsequently adding them to the existing Rete node structure. In this study, an existing Rete network was implemented using the Boost C++ library, and a hash index for the CQ space was implemented as an R* tree. We designed and executed a minimum bound rectangle (MBR)-based hash index for all spatiotemporal conditions of the input rules. The MBR, expressed by transforming the spatial nodes of the rule, contained the minimum rectangle of the spatial nodes and the rule type. The moving object type of the enemy ship rule is ship, which we will call a rule type. The rule type of the enemy aircraft rule is aircraft. The index for the CQ area is made as a CQ index separated by rule type.

**Figure 6:**
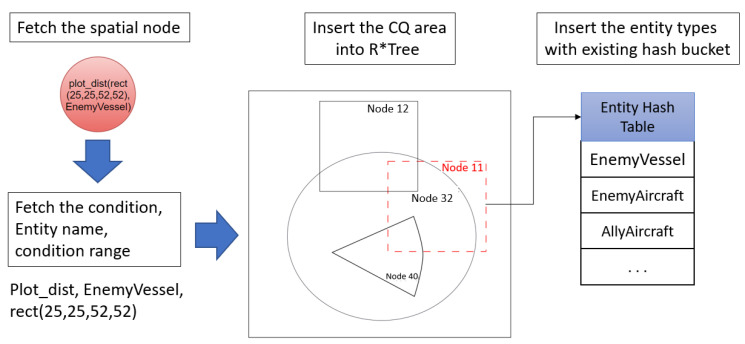
Inserting a spatial node into an index.

The CQ index separated by rule type was implemented using R* tree using the MBR of each CQ. The location of the target objects is used as the hash key of the CQ index. In Figure 6, the newly inserted MBR of Node 11 is added to the R* tree and bound to the entity hash table.

A spatial node referred to as ‘plot_dist(rect(25,25,52,52) EnemyVessel’ was created from the rules described in the text. Node 11 (red dotted square in Figure 6), representing the MBR of the spatial node, was inserted into the R* tree. By inserting the entity of the spatial node into the entity hash table by type, the index and hash table were connected. For example, as shown in Figure 6, there was ‘plot_dist( )’ in the spatial node of the rule; thus, the corresponding MBR was inserted into the R* tree. Since these rules only applied to events for ‘EnemyVessel’, stabbing the MBR node was added from the R* tree points to the hash bucket of the ‘Entity Hash Table’. The CQ index and hashing bucket process for processing spatial nodes in the Rete algorithm are described in Algorithm 1.

### 3.2. Stabbing Algorithm for Continuous Query Events

Stabbing Rete nodes for continuous spatiotemporal queries is a method used to determine the rules that need to be applied to incoming events with spatiotemporal indexes. The stabbing procedure for nodes for an incoming event in this study is as follows. First, the target object event coming into the data distribution service topic was treated sequentially in the working memory, which managed all external events entered prior to the starting of the Rete network. Second, it was examined against the alpha node of all rules that did not include a sliding window for the highest priority event in the working memory. Third, we examined an alpha node with a temporal condition. A rule with a time condition was executed at the trigger time, which is the point where the node condition was checked for the event contained in the buffer.

**Figure 7:**
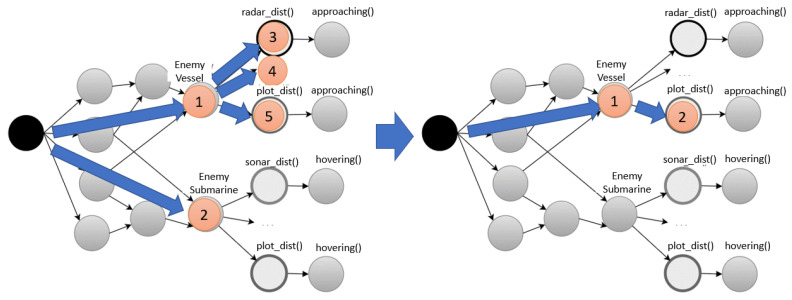
Hash indexing for beta nodes.

As can be observed in Figure 7, when there was no hash index for the ‘EnemyVessel’ beta node, alpha nodes 3, 4, and 5 were examined consecutively. When a hash index was created, it was directly stabbed to alpha node 2 with respect to beta node 1. Since a hash index was used, all events were classified using buckets in the hash table, indicating the entity type of each event. The node-stabbing process using the hash index was implemented based on Algorithm 2. Node stabbing was processed with intersect () for the event location, as shown in Algorithm 2.
**Algorithm 2** Event Stabbing (e)1  **Input:** Event //Tested event with its attributes; 2  **Output:** List<String> //Result of Event evaluation;   /* Copy the input into Working Memory queue */ 3  mainWM.pushEvent(Event);  /* stab the event */ 4  If (IsNotEmpty(mainWM)) {  /* Define the entity hash */ 5  List<Node*> stabbed; 6  While (ScalarNode != NULL) { 7   If (ScalarNode[i].test(Event)) { 8    stabbed.push(ScalarNode-i); 9   } 10  } 11  EntityHash = EntityNodeList[stabbed]; 12  EntityNode.pushEvent(Event);  /* spatial node stabbing */ 13  *SpatialTree = SpatialIndex[EntityHash]; 14  *root = *SpatialTree; 15  EventLocation<int, int> = {Event.Latitude, Event.Longitude}; 16  List<Node*> stabbedCQ; 17  While (*root != NULL) { 18   If (IsALeaf(*root)) { 19    stabbedCQ.push(*root); 20    *root = NULL; 21   } 22   Else { 23    For (auto *leaf in *root.leaf) { 24      If (Intersects(EventLocation, *leaf) == true) { 25       *root = *leaf; 26       break; 27      } 28     } 29   }   /* collect the result in a list of string */ 30   List<string> res; 31   For (auto s in stabbedCQ) { 32    s.push(Event); 33    res.Append(Node.evaluate()); 34   } 35   return res; 36  } 37 } 38 Else { 39  continue; 40 }

Stabbing events based on entity types using hash indices were *O*(1). Events did not require individual execution for every rule because we used the CQ node index. The existing Rete algorithm was *O*(*n*) when there were n CQ nodes, whereas stabbing by the hash index was processed as *O*(*log n*).

## 4. Performance Evaluation

We present a scenario for a performance evaluation test using Drool. The performances of the two methods changed when the number of rules and the number of target objects to be examined increased.

### 4.1. Hashing Index of Rete Node

The performance comparison experiment was performed on a PC using a Windows 10 64-bit operating system, Intel i5 3.2 GHz 64-bit processor, 16 GB memory, and Visual Studio C++. A virtual target dataset was created and tested because access to real data was impossible owing to military security. For the properties of artificially generated events, the range of properties, data types, and values were set, as shown in Table 1.

The test scenario was established for the following three factors: First, the number of rules was varied, and various types of rules were tested. Second, different types of target objects were tested by varying the number of target objects. Third, a performance test was carried out according to the change in movement patterns and types of target objects. The CQ area expressed in the rule used a radar-based circle, a sonar-based sector, and a square of a hazardous area. Table 2 shows a scenario in which the ratio of radar, sonar, and square was different for the number of rules from 50 to 100.

For the second factor influencing the performance test evaluation, test scenarios were created according to the distribution and types of target objects. Target objects were classified into air, water, and underwater objects, and the uniform distribution and non-uniform distribution of each object were tested. In the case of non-uniform distribution, air, water, and underwater were set to 60%, 20%, and 20%, respectively. Table 3 shows that the possible range of speed, flight altitude or depth of water, and enemy and ally are different depending on the entity type.

**Figure 8:**
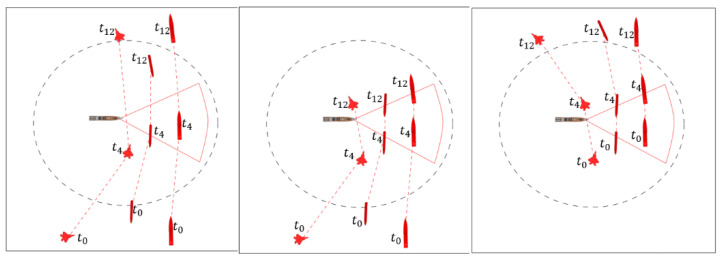
Scenarios of movement patterns of target objects.

The third factor in the performance comparison experiment is the movement pattern of the target objects. In order to measure the rule processing performance for a given CQ, a meaningful tactical movement pattern, not a random movement pattern, is targeted. Representative movement patterns of target objects were tested for approach, detour, U-turn, and turning. In addition, as a movement path scenario combining basic movement patterns, the U-turn after approach, detour after approach, and turn after approach scenarios were also considered. Figure 8 shows the movement pattern scenario for the sectoral CQ region. In addition to the sectoral CQ, test evaluation scenarios were created for the circular CQ and the rectangular CQ.

### 4.2. Performance Testing on the Number of Rules and the Number of Target Objects

**Figure 9:**
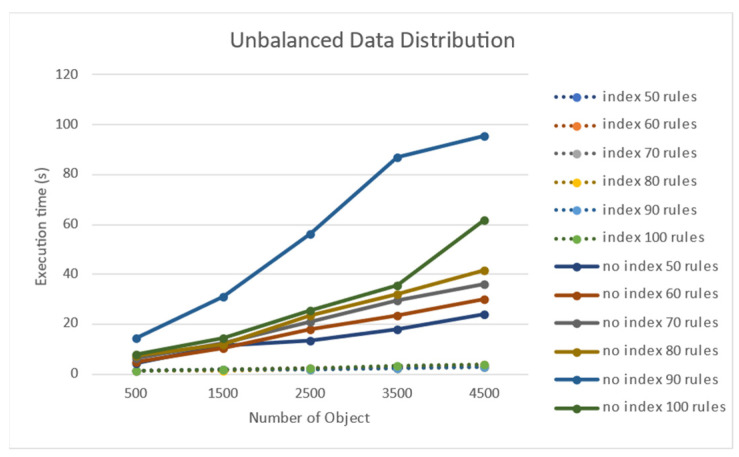
Performance effect of the number of objects on a non-uniform object distribution.

When the target objects were non-uniformly distributed in a two-dimensional space, a performance test was conducted. When the number of rules increased, there was a negligible change in the processing time of the rules when using indexes for Rete nodes. Nonetheless, when the node index was not used, the processing time increased as the number of target objects increased. Figure 9 shows that the processing time increases when the number of non-uniform target objects increases. The execution time on the y-axis is in seconds (s), and it took 60 s for 4500 targets for 100 rules when no node index was used.

**Figure 10:**
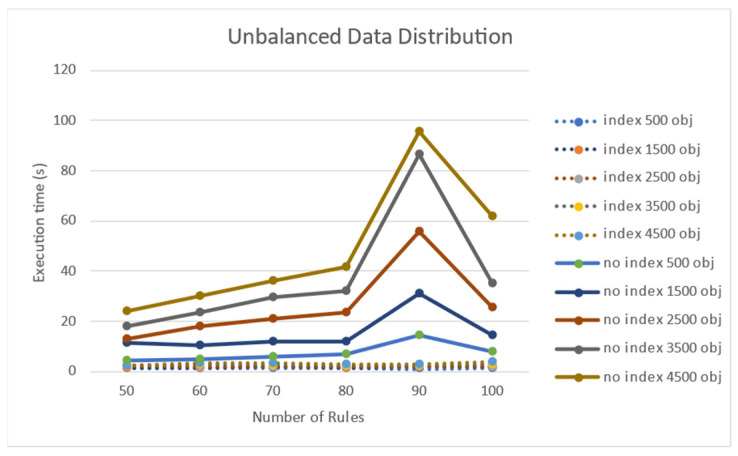
Performance effect of the number of rules on a non-uniform object distribution.

Figure 10 shows the experimental results for the performance effect of the number of rules for the case of non-uniformly distributed target objects. When a node index was used, an increase in the number of rules did not significantly affect the performance. However, when the number of rules increased in the absence of a node index, the performance significantly deteriorated.

**Figure 11:**
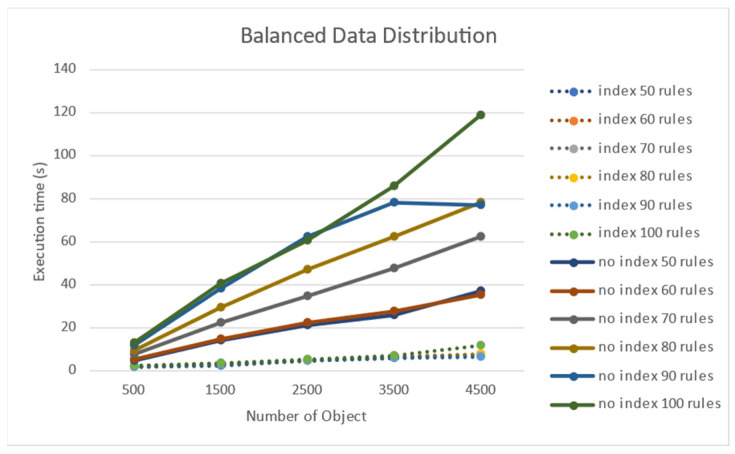
Performance effect of the number of objects on a uniform object distribution.

**Figure 12:**
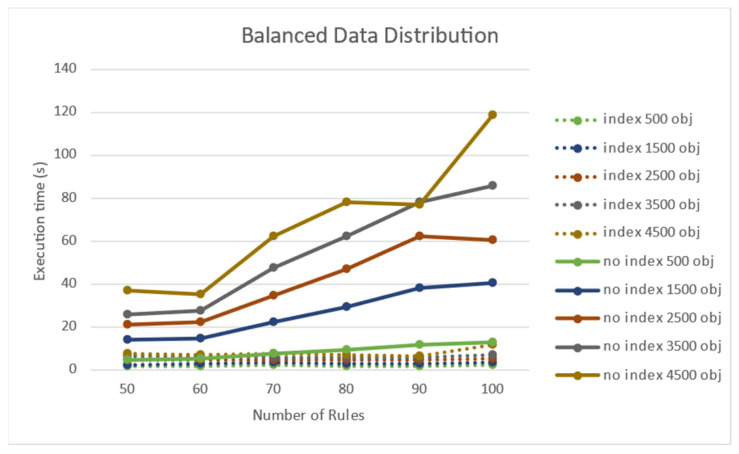
Performance effect of the number of rules on a uniform object distribution.

Figure 11 shows the experimental results of the performance effect of the number of target objects when the target objects were evenly distributed. When the distribution was uniform, the processing time was longer as the number of objects increased. Figure 12 shows that rule processing time becomes longer when the number of rules increases uniformly in the absence of a node index.

The results of the performance evaluation experiment showed that the method that used the index for the Rete node was reduced to a log function compared to the method that did not use the index. Let the number of target objects be N, the number of nodes in the Rete network be V, and the number of links be E. When the index for the Rete node is not used, the time complexity of the stabbing algorithm for CQ is expressed as O(n* (|V| + |E|)). When creating an index for CQ in the Rete network, the stabbing algorithm is described as O(log_2_(n* (|V| + |E|))) because it follows the time complexity of R* tree.

### 4.3. Performance Comparison Test with Drool

A performance comparison evaluation experiment was conducted with Drool, which is a commercial rule-processing tool implemented based on the Rete algorithm. A performance comparison experiment was performed on the same rules and target objects. A comparative experiment was conducted to measure processing time for Drool and the Rete node indexing technique presented in this paper. The measurement time in Table 4 is in milliseconds, and the performance improvement rate is calculated as:

*Rate* = (*TRete* − *TModifiedRete*)/*TModifiedRete* × 100%. (1)

As an example of the processing time performance improvement rate improvement method, if 100 s is reduced to 50 s, it is calculated as (100 − 50)/50 = 100%.

**Figure 13:**
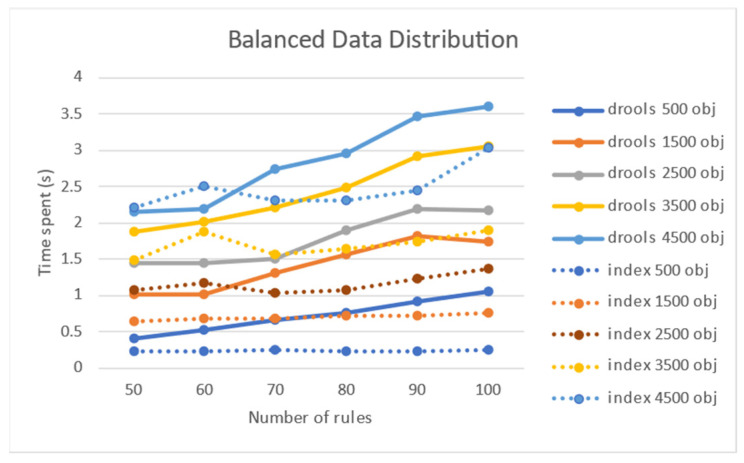
Performance comparison with Drool according to the number of rules.

The increase in the rule processing time when the distribution of target objects was uniform is significant compared to when the distribution was non-uniform. A comparison with Drool was measured solely for a uniform distribution. The processing time of Drool required more than 3.5 s when 100 rules and 4500 target objects were used. As shown in Figure 13, the node index method presented in this study, compared to that of Drool, showed superior performance when the number of objects was small. When the number of rules was 100, the node index method achieved a performance improvement of 329%, 59%, and 18% for 500, 2500, and 4500 objects, respectively.

**Figure 14:**
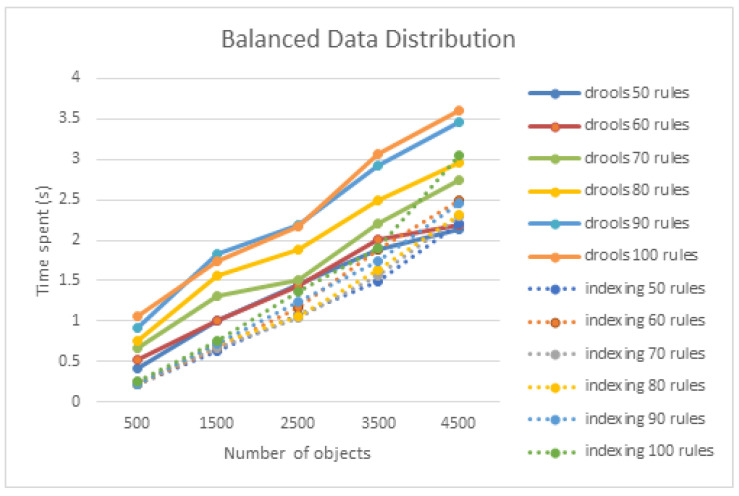
Performance effect of the number of objects on uniform object distribution compared to Drool.

When the target objects were evenly distributed in the performance comparison experiment with Drool, the performance effect of the number of rules was lower than the effect of the number of target objects. As can be observed in Figure 14, when the number of target objects is 4500 for 100 rules, Drool exceeds 3.5 s, and the node index method requires more than 3 s. Based on the experimental results, the increase in the number of rules had an insignificant effect on performance. This may be attributed to the non-application of the compiled structure and index of the rules. Nonetheless, it was concluded that an increase in the number of target objects had a greater impact on performance because it appeared as an increase in processing load, whether it was a Drool or a node index.

## 5. Conclusions

When there are many CQ rules with spatiotemporal conditions and many target objects input in real time, the Rete method for rule processing also has a problem with poor performance. In order to solve the overhead of searching for spatiotemporal CQs one by one in the Rete node link structure, a hash index technique for spatiotemporal CQ is proposed. For the proposed spatiotemporal CQ index, a CQ index with a hash bucket bound to an entity type is created. It has been demonstrated that the implementation of node indexing can reduce the rule search overhead by filtering out beta events that are unrelated to the continuous query space.

The execution times of the node indexing method proposed in this study and the existing tool (Drool) were measured by varying the number of rules and the number of target objects when the target objects were evenly distributed. When 100 rules were used, the performance improvement rate was 329% and 18% when the number of objects was 500 and 4500, respectively. The performance improvement rate of the node index method did not exceed that of Drool when the number of objects increased. This is because Drool is an optimized tool proven to offer excellent performance when the number of objects is substantial.

In the future, when the number of objects exceeds 4000, additional performance improvement will be required by introducing object indexing and buffering of objects by rule. Next, the effectiveness of the spatiotemporal CQ index on the uncertainty of dynamically input real-time events should be verified, and the complex mass function [33] should be applied to determine the interference effects.

## Figures and Tables

**Table 1:** Object initialization parameters.

No.	Attribute	Data Type	Value	Meaning
1	Event Id	Integer	1–∞	Event id
2	Time	Long Long	0–∞	Event time
3	Speed	Float	−30–200	Object speed (m/s)
4	Elevation	Float	0–10	Object elevation (10 m)
5	IFF	Boolean	True, False	Friend or Foe
6	Latitude	Float	115–136	Latitude location
7	Longitude	Float	15–46	Longitude location
8	Object Type	String	fighter, destroyer, etc.	Object type or mode
9	Object Id	Integer	∞	Object id of current event

**Table 2:** Scenarios according to the number and type of rules.

No.	Rules	Radar	Sonar	Plot Dist
1	50	34%	32%	34%
2	60	41%	28%	30%
3	70	44%	27%	28%
4	80	43%	25%	31%
5	90	42%	24%	33%
6	100	25%	25%	50%

**Table 3:** Speed, altitude, and IFF ranges of target objects.

No.	Entity Type	Speed (m/s)	Elevation (m)	IFF
1	Enemy Aircraft	10–100	10–100	FALSE
2	Ally Aircraft	10–100	10–100	TRUE
3	Enemy Vessel	3–100	0–10	FALSE
4	Ally Vessel	3–100	0–10	TRUE
6	Enemy Submarine	0–100	−10–0	FALSE
7	Ally Submarine	0–100	−10–0	TRUE

**Table 4:** Performance improvement ratios for uniform distribution of target objects.

Number of Rule Nodes	Objects	Average Time (ms)		Improvement Rate
		Indexed Rete	Original	
50	500	220	409	85%
	2500	1.071	1.448	35%
	4500	2.143	2.211	3%
60	500	226	521	130%
	2500	1.163	1.444	24%
	4500	2.183	2.500	14%
70	500	245	662	170%
	2500	1.041	1.512	45%
	4500	2.303	2.736	18%
80	500	222	762	243%
	2500	1.061	1.893	78%
	4500	1.631	2.958	81%
90	500	226	913	303%
	2500	1.237	2.183	76%
	4500	2.450	3.463	45%
100	500	246	1.057	329%
	2500	1356	2.169	59%
	4500	3.042	3.599	18%

## Data Availability

As a result of military security concern, we can only present the artificially generated data in Figshare at https://doi.org/10.6084/m9.figshare.16817635.
